# Granular flow experiment using artificial gravity generator at International Space Station

**DOI:** 10.1038/s41526-023-00308-w

**Published:** 2023-08-08

**Authors:** S. Ozaki, G. Ishigami, M. Otsuki, H. Miyamoto, K. Wada, Y. Watanabe, T. Nishino, H. Kojima, K. Soda, Y. Nakao, M. Sutoh, T. Maeda, T. Kobayashi

**Affiliations:** 1 Yokohama National University, Yokohama, Japan; 2 Keio University, Yokohama, Japan; 3 Japan Aerospace Exploration Agency, Sagamihara, Japan; 4 The University of Tokyo, Tokyo, Japan; 5 Chiba Institute of Technology, Chiba, Japan; 6 Tokyo University of Agriculture and Technology, Fuchu, Japan; 7 Ritsumeikan University, Kusatsu, Japan

**Keywords:** Aerospace engineering, Mechanical engineering

## Abstract

Studying the gravity-dependent characteristics of regolith, fine-grained granular media covering extra-terrestrial bodies is essential for the reliable design and analysis of landers and rovers for space exploration. In this study, we propose an experimental approach to examine a granular flow under stable artificial gravity conditions for a long duration generated by a centrifuge at the International Space Station. We also perform a discrete element simulation of the granular flow in both artificial and natural gravity environments. The simulation results verify that the granular flows in artificial and natural gravity are consistent. Further, regression analysis of the experimental results reveals that the mass flow rate of granular flow quantitatively follows a well-known physics-based law with some deviations under low-gravity conditions, implying that the bulk density of the granular media decreases with gravity. This insight also indicates that the bulk density considered in simulation studies of space probes under low-gravity conditions needs to be tuned for their reliable design and analysis.

## Introduction

Technologies for landing and roving on extra-terrestrial bodies are fundamental for pursuing scientific missions and human exploration in space, as reported in the roadmaps published by several space agencies^1–3^. Solar rocky bodies, including the Moon and asteroids, are covered by fragmental debris called regolith. Regolith, which is a potentially fluffy and powdery granular material, is a primary concern for the lander or rover; landing on such loose soil is a critical phase during exploration as the footpad of the landing gear may bury into the regolith. Therefore, the mechanical interaction of the footpad plays a key role in the landing dynamics^4–6^. The importance thereof is borne out by the fact that one mission involving Spirit, one of the twin Mars exploration rovers, had to be terminated because the wheel of the rover were trapped by loose regolith^7^. This issue has clarified the importance of the wheel–soil interaction mechanics^8,9^. These mechanical interaction models of machines (i.e., landing gear or mobility system) on such granular media under various levels of gravitational acceleration are key to their reliable design and analysis. For instance, the pressure–sinkage relationship^10^, often seen in a wheel–soil interaction model, considers the weight density of granular media for determining sinkage with respect to the pressure applied. The alternative models proposed in the last decade, such as the empirical resistive force theory^11–13^, a local friction force model^14^, a unified force model for the drag^15^, and a modified Archimedes law^16^, also implicitly include gravitational acceleration because they assume hydrostatic-like force in accordance with the penetration depth of solid objects into granular media.

The discrete element method (DEM) is a numerical approach that precisely simulates the dynamic response of granular media in mechanical interaction^17,18^. In the past, several studies using DEM focused on predicting vehicle mobility in regolith sediments on the Earth, Moon, or Mars^19–23^ as they explored the effect of gravity on sinkage and motion resistance of vehicle wheels. The DEM has also been employed to address granular media in low-gravity environments^19,20,24^.

Such a computer-aided approach often requires a reliable scheme for the accuracy evaluation of the simulated output. According to verification-and-validation (V&V) standards^25,26^, any simulation model and its output must be quantitatively evaluated and validated by comparing them with the corresponding experimental results. Therefore, the DEM analysis of granular dynamics in an extra-terrestrial environment also needs benchmark data obtained at the corresponding test environment under varied gravity and high-vacuum conditions to be verified.

The mechanical characteristics of granular media under different gravity conditions have been experimentally investigated, and it was found that their flow dynamics explicitly depend on the gravitational acceleration^27^. The angle of repose is constant regardless of the gravitational acceleration^28,29^, while the friction angle slightly increases as the gravity decreases^30^. The wheel mobility performance under reduced gravity has also been investigated^31^. The abovementioned studies^27–31^ employed parabolic flights or drop tower facilities for simulating reduced gravity environments on Earth. These facilities, however, can simulate only limited campaigns: short duration (e.g., a few tens of seconds in parabolic flights), fewer test trials, and unstable gravity conditions^32^.

While the experimental approach limits the number of trials and gravity conditions, dimensional analysis is a typical approach used for discussing the gravity-dependent characteristics of the granular flow. Beverloo’s law^33^, the most commonly used law for flow rate analysis of granular media, postulates that the steady-state mass flow rate is proportional to the square root of the gravitational acceleration. The law has been modified for wider outlet sizes^34^ and validated under high-gravity conditions^35–37^. However, its applicability under low-gravity conditions is still uncertain because of the lack of experimental data under the low-gravity condition as noted above.

The abovementioned discussion clarifies the scientific purpose of this study: to propose an experimental approach to examine a granular flow under stable artificial gravity conditions for a long duration, to reveal the gravity-dependent characteristics of granular flow under low-gravity conditions, and to examine the characteristics according to the mechanical phenomena and Beverloo’s law. Further, publicly available data obtained in the low-gravity experiment may be scientifically beneficial for the research community. Therefore, we conducted granular flow experiments under low-gravity conditions by using hourglass-shaped devices in the Japanese Experimental Module (JEM) at the International Space Station (ISS). The JEM is equipped with a centrifuge providing high-quality, long-term, and stable artificial gravity (AG) conditions^38,39^. We employed a wide range of AG levels, from 0.063 *G* to 2.0 *G*, over seven hours using several granular media. A DEM simulation study also supported the consistency of the granular dynamics in the AG and natural gravity fields. We found that the mass flow rate of the granular media measured at the experiments quantitatively follows Beverloo’s law, while some deviations are found under low-gravity conditions. The statistical and dimensional analysis of the deviation implies that the low-gravity decreases the bulk density of the granular media.

Our experiments provide further insights for future experiments on granular media using an artificial gravity generator and/or hopper-shaped experimental apparatus. The result of the mass flow rate is a valuable benchmark for data correlation or assimilation for future experiments. Additionally, the size of the orifice diameter relative to the particle diameter, the amount of granular media packed in the hopper, and camera settings (such as image resolution, frame rate, or field of view) are important for developing a similar experimental test bench.

There are several lunar/planetary exploration missions planned for the coming decades: CLPS (Commercial Lunar Payload Services) offered by NASA accelerates the lunar exploration and in situ resource utilisation as part of the Artemis programme where several companies shall deliver landers and rovers to the moon. The Mars sample return mission requires soft and accurate landing and fast roving on Mars. The Martian moons exploration (MMX) by JAXA also requires a high-fidelity landing simulation under low-gravity conditions (approximately 0.058 *G*). Our results can be used to improve dynamic analyses using numerical simulations for these landers/rovers by tuning the bulk density to lower values. Without the tuning of the bulk density, existing simulation techniques overestimate the traction performance of the rover and underestimate the sinking of the landing footpad, whereas improved simulations based on our findings could result in more accurate values for these dynamic analyses. This outlook provides useful insights into the design and analysis of space probes under low-gravity conditions.

## Results and discussion

### Experiment

**Fig. 1:**
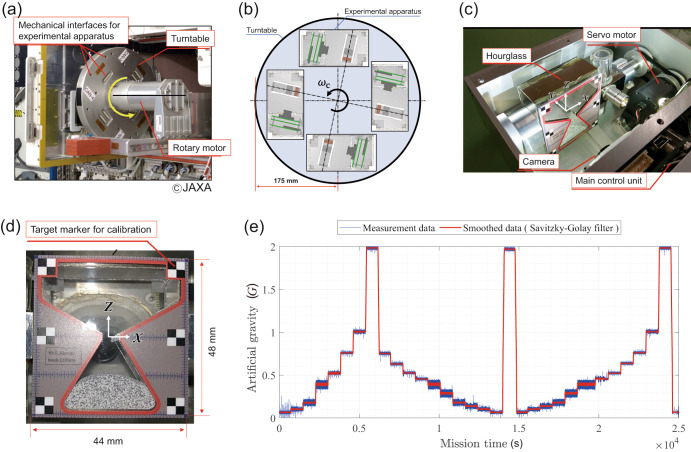
Overview of the experimental setup, apparatus, and time history of the AG profiles. **a** Centrifuge installed on the JEM/ISS (courtesy JAXA/NASA) without attaching the experimental apparatus; **b** Schematic illustration of the centrifuge with the experimental apparatus. The centrifuge installs four sets of the apparatus in each experimental run. We performed two experimental runs for eight sets in total; **c** Snapshot of the experimental apparatus (top cover is removed). The reference coordinate of the hourglass is located at the centre of the orifice; **d** Hourglass image taken by the camera (trimmed image); and **e** Time history of AG in the experimental run ranging over the values 0.063, 0.10, 0.17, 0.38, 0.50, 0.75, 1.0, and 2.0 *G*.

The centrifuge (Fig. 1a, b) in the experiment is used to rotate the experimental apparatus (Fig. 1c, d) with arbitrary angular velocity to obtain AG range of 0.063–2.0 *G* with specific gravity steps every 13 min (Fig. 1e). We developed a total of eight sets of the experimental apparatus, each containing different granular media in the hourglass-shaped hopper. Table 1 summarises the type of granular media along with the mass packed, microphotograph, and snapshots after the flow under microgravity (0.063 *G*). Note that the inside of each hourglass is vacuum. The typical properties of the granular media, such as particle size distribution, shear strength, and particle and bulk densities, are available in Supplementary Methods. The experimental campaign continued for seven hours. Each hourglass continued flipping every 60 s to change the flow direction to different hopper angles (60° and 120°). The total number of flipping was more than 400 during the experimental campaign. Supplementary Methods describes the geometric layout of the experimental apparatus, specifications, and the angular velocity of the centrifuge according to varied AG levels. The acceleration profile of each AG is also summarised in Supplementary Methods. The Data Availability section provides the raw movies (MP4 files) of the flows of the granular media.

**Table 1:** Snapshots of granular materials in microgravity. The hourglass image represents the final state of the flow when the granular media flows from the narrow area to the wide area (hopper angle is 60°) in 0.063 *G*. The mass of the granular media *m* summarised in Table 1 is determined by the following equation: *m* = *V*_60_ *ρ*_*min*_, where *V*_60_ represents 60 % of the total volume of the narrower space of the hopper (the side of the hopper angle being 60 degrees), and *ρ*_*min*_ is the minimum bulk density of the granular media (Supplementary Table 1). The value of *V*_60_ (60 %) is conservatively determined to avoid the overflow of the granular media.

Granular material (Mass packed in the hourglass)	Microphotograph	Hourglass image
#01 Alumina beads (6.72 ± 0.01 g)		
#02 Silica sand No. 5 (Tohoku sand) (4.66 ± 0.01 g)		
#03 Toyoura sand (4.38 ± 0.01 g)		
#04 Lunar regolith simulant (FJS-1, sieved) (4.76 ± 0.01 g)		
Granular material (Mass packed in the hourglass)	Microphotograph	Hourglass image
#05 Phobos regolith simulant (3.35 ± 0.01 g)		
#06 Martian regolith simulant (JSC MARS-1A) (2.36 ± 0.01 g)		
#07 Silica sand No. 8 (Tohoku sand) (3.71 ± 0.01 g)		
#08 Lunar regolith simulant (FJS-1) (4.93 ± 0.01 g)		

Among the granular media listed in Table 1, the alumina beads, silica sand No. 5, and Toyoura sand exhibited mass flow with some periodic intermittency effects^40^, particularly observed under the condition of relatively low-gravity (see Data Availability section). The intermittency is due to the partially generated arch above the orifice^40^, which is formed by the solid friction forces between the particles and/or walls but never leads to permanent clogging. The arch collapses as the partial flow continues because the bearing force at the arch generated between the particles becomes smaller than the arch weights (total particle mass times gravity), and the stick-slip effect occurs between the particles^40^.

By contrast, the other materials flew with multiple modes such as mass/funnel flows^41–43^ that varied with the levels of gravity conditions, and they also expressed bridging and rat hole. In addition, these granular media adhered, deposited, or even aggregated because of their wide particle size distributions and non-spherical particle shapes, generating the interlocking force between the particles.

**Fig. 2:**
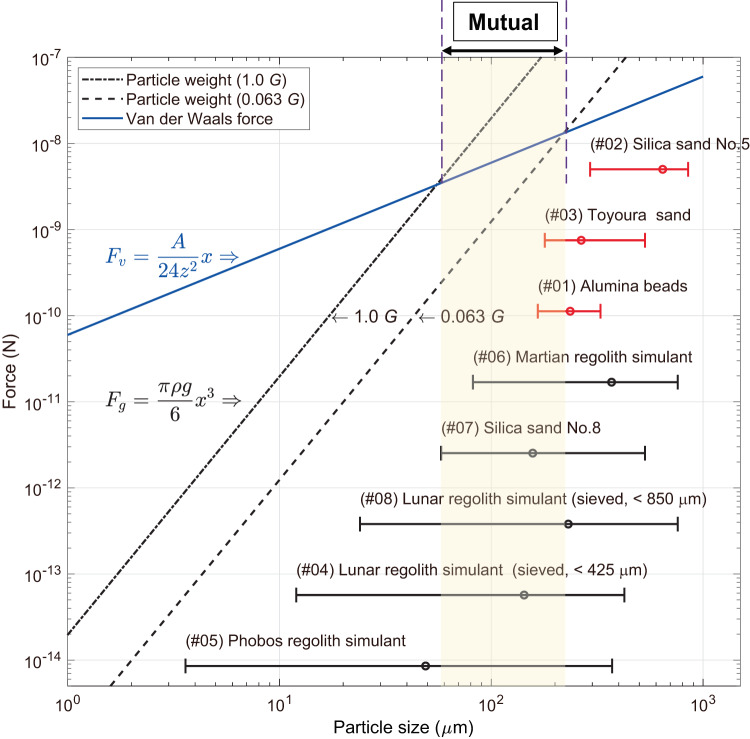
Relationship of particle size *x*, particle weight, and Van der Waals force. The particle is assumed to have a spherical shape and density *ρ* for the evaluation of forces. The Van der Waals force is estimated under assumptions of the alumina composition without the surface roughness. The parameters of the Van der Waals force are as follows: Hamaker constant *A* = 15.5 × 10^−20^ J, and *z* = 10 nm. The horizontal error bars in the graph show the range of particle sizes of each tested granular media. The open-circle is the median diameter, and both ends indicate the minimum and maximum particle ranges. The vertical location of the error bars is arbitrary.

Figure 2 illustrates the relationship between the particle size and gravitational forces (particle weight) and Van der Waals force of a particle. The median diameter (D50) of the granular media is indicated as the open-circle plot along with the minimum and maximum particle range of the granular media. Assuming a spherical particle, the effective particle weight due to gravity is proportional to the cube of the particle size *x*. In contrast, the Van der Waals force (alumina with a smooth surface) is proportional to *x*.

Figure 2 illustrates that the Van der Waals force generated at the particle size smaller than approximately 50 *μ*m becomes larger than the particle weight owing to gravity even under 1.0 *G*. In particular, the granular media (#04 to #08, Table 1) contain a large number of fine particles, resulting in a remarkable effect of adhesive forces. Furthermore, because of the repeated flow by flipping the hourglass, the fine particles collide with one other or with the walls under vacuum conditions. An electrostatic force may be generated owing to the charged particles, which is beyond the scope of this study. Nevertheless, we deduce that this force may excite the particle adhesion to the hourglass wall made of borosilicate glass. These factors impede the explicit flow of the granular media of #04 to #08, as shown in the snapshots of Table 1.

In contrast, the particle weight owing to gravity for particle size larger than approximately 200 *μ*m becomes larger than the Van der Waals force even under microgravity (0.063 *G*). The granular media, alumina beads, silica sand No. 5, and Toyoura sand are above this threshold and have a relatively low internal friction angle (see soil property in the Data Availability section), showing explicit granular flows under varying gravity levels. Thus, the following analysis focuses on these three granular media to discuss the gravity-dependent characteristics of granular flow.

**Fig. 3:**
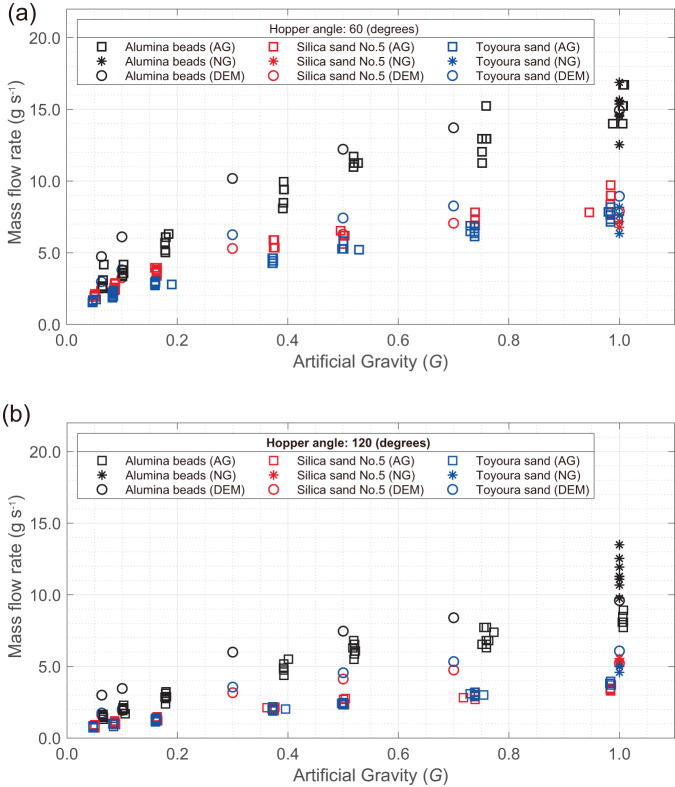
Mass flow rates of granular media in varied AG levels. The results were obtained for hopper angles of **a** 60° and **b** 120°. The mass flow calculated from DEM in AG environment and tested in NG (1.0 *G*) environment are plotted with the open-circle and asterisk, respectively. The flowing time (mass divided by the mass flow rate) was 0.40 s at the shortest (Alumina beads at 1.0 *G*) and 6.56 s at the longest (Silica sand No. 5 at 0.063 *G*).

Figure 3 shows time-averaged values of the mass flow rates during flow under different AG levels. The mass flow rates of all granular media are proportional to a power law of the AG, regardless of the hopper angles. The granular flows at the hopper angle of 60° are faster than those at 120°. This is because the steeper the slope is toward the hourglass orifice, the higher is the flow rate^44^. Among the three granular media, the alumina beads have a relatively high mass flow rate because of their spherical particle shapes with uniform diameters. The flow rate of silica sand No. 5 and Toyoura sand are almost equivalent, although their particle sizes and distributions are different. This is discussed in Statistical analysis section based on Beverloos’s law.

Figure 3 also includes the mass flow rate measured during pre-flight experiments on Earth, under the natural gravity (NG) condition of 1.0 *G*. At the hopper angle of 60°, the granular flow under NG is almost unchanged within the range of temporal resolution of the camera used. The mass flow rate at the hopper angle of 120° under NG is faster than that under AG. This may be due to the uniform gravitational field applied by NG to the granular media even after passing through the orifice. On the other hand, the gravity in the AG is inversely proportional to the rotational radius of the apparatus (see Supplementary Methods). Moreover, the granular media passing through the orifice is subject to the constant velocity motion. The DEM-based analysis on the flow modes examines the difference between the AG and NG conditions, as described in the following section.

### Discrete element method analysis

The DEM analysis was performed to examine the difference between the AG environment at the ISS and the NG environment. In the former, the experiment at the ISS was mimicked, i.e., the hourglass was repeatedly flipped while being revolved (see movies 1 and 2 in Supplementary Notes). However, in the latter, a prescribed uniform gravitational field was applied to granular media, and only repeated flipping of the hourglass was performed.

**Fig. 4:**
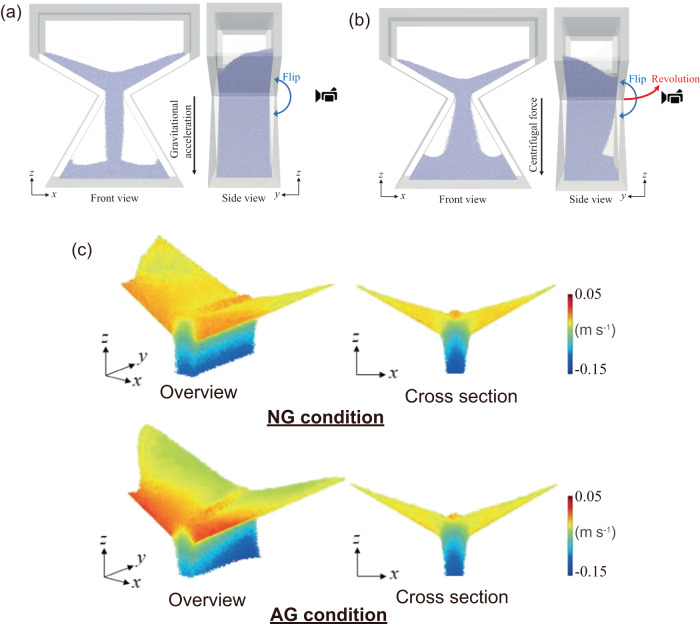
Snapshots of DEM analysis results of alumina beads at 0.1 *G*. **a** NG environment; **b** AG environment in the ISS. The front view of the hourglass corresponds to the right-hand side in the side view; **c** Colour map of particle velocity fields in the upper box of hourglass. The magnitude of velocity is in the direction of gravity/centrifugal force.

Figure 4a, b shows a snapshot captured during the flow of alumina beads under 0.1 *G*. Details on the DEM parameters and conditions are provided in the Methods section. It can be confirmed that in the AG environment, the particles fall towards the rearward direction deviating from the centrifugal force direction after passing through the orifice. The reason is that the falling particles are in the inertial coordinate system, and when observing them from the hourglass in the rotating coordinate system, the Coriolis force induces particles to move in the tangential direction of the rotating orbital. The granular flow therefore deviates towards the +*y* direction (thickness direction) of the hourglass. However, for AG, the sedimentary particles in the upper section of the hourglass are in the rotating system just like the hourglass. Figure 4c shows the colour map of particle velocity fields in the upper box under AG and NG conditions. In the case of mass flow mode, it can be confirmed that the velocity fields in the direction of gravity/centrifugal force are similar regardless of AG and NG environments. In particular, the arch-shaped distributions around the orifice, which are related to the flow rate, are consistent with each other. Therefore, the effects of gravity condition on the mass flow in the upper box are implied to be similar to those of NG.

Figure 3 also includes the gravity dependence of the mass flow rate obtained via the DEM analysis for alumina beads, silica sand No. 5, and Toyoura sand. Furthermore, the DEM analytical results with respect to the trend of gravity dependence of mass flow rate, the relative magnitude of the mass flow rates among these three granular materials observed in the experiment, and the relationship calculated by DEM are in good agreement with experimental results. Thus, we could examine the effects of the gravitational environment on the flow rate. Additionally, we compared the granular flow of alumina beads obtained from the experiment on Earth (1.0 *G* under NG) and from the DEM analysis (1.0 *G* under NG), as shown in the Supplementary Notes. It was confirmed that the granular flow behaviour under 1.0 *G* (NG) was well reproduced under the present conditions of DEM analysis.

**Fig. 5:**
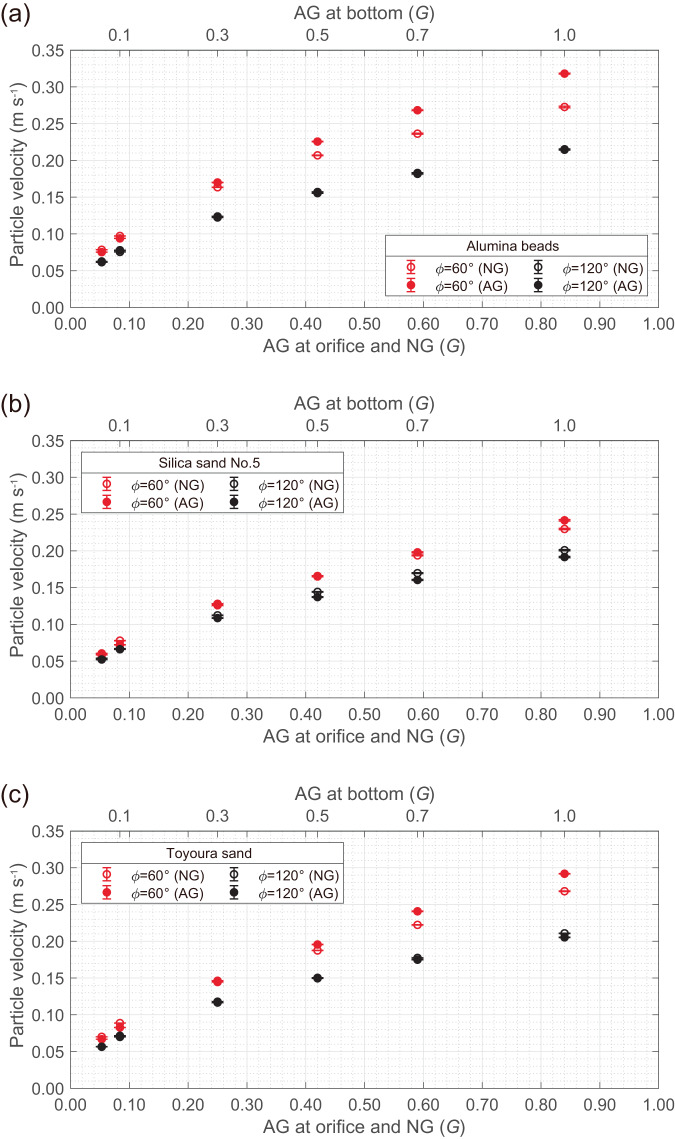
Gravity dependence of particle velocity just below the orifice obtained by DEM analysis under NG and AG conditions. **a** Alumina beads; **b** Silica sand No. 5; and **c** Toyoura sand. The particle velocity was measured at the centre position immediately below the orifice (*x*-*y* plane with 5 mm ×15 mm, and 1 mm length in the *z* direction). The error bar denotes 95%CI. The measurement method and measured data of particle velocity are available in Supplementary Methods.

Figure 5 shows the gravity dependence of the particle velocity obtained by DEM analyses for alumina beads, silica sand No. 5, and Toyoura sand. Here, the particle velocity was measured at the central position just below the orifice and was evaluated as the averaged velocities of passing particles. For the detail of the measured data, see Supplementary Methods. The open and closed plots correspond to the NG and AG conditions, respectively. Because the effect of the Coriolis force on the flow rate immediately after the fall is small (falling distance is less than 1 mm), the results of gravity-dependency are almost the same under both gravity conditions, especially in the case of a hopper angle of 120° or of low-gravity level. Meanwhile, when the hopper angle is 60° and a high-gravity level is employed, the magnitude of the average velocities is different between the NG and AG conditions. However, it is noteworthy that the average particle velocities of all the granular media are still proportional to the square root of the gravity regardless of the gravity conditions.

**Fig. 6:**
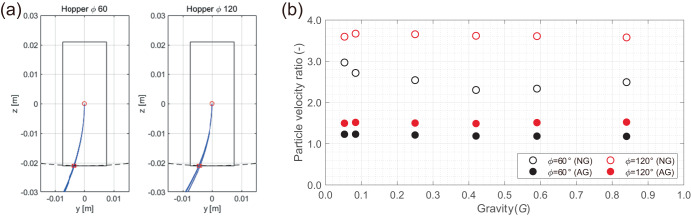
Particle motion of the alumina beads obtained by the equation of motion. **a** trajectories of the particles as seen from the *y-z* plane of the hourglass under each AG condition; **b** ratio of the velocities at the bottom to the initial velocity. DEM analysis results are used for the initial velocities of particles just below the orifice. The solid symbol represents the data for the AG, whereas the open symbol represents that for the NG.

Figure 6a shows the calculation results of the trajectories of the particles as seen from the *y-z* plane of the hourglass under the AG condition. Here, we used the initial velocities of the particles obtained via the DEM analyses under the AG condition (Fig. 5) as those for trajectory calculation. Because the angular velocity of the rotary table changes according to the magnitude of the AG, similar trajectories are seen regardless of the AG level within the range of geometric conditions of the present experiment. The particles on the camera side do not collide with the back wall during the fall (Fig. 4).

Figure 6b shows the ratio between the velocities at the bottom collision to the initial falling velocity immediately below the orifice. The magnitude of the velocities at the bottom was evaluated by considering a constant acceleration motion in the NG environment and revolution condition in the AG environment. Here, the maximum drop height in the hourglasses is 21 mm (see Supplementary Fig. 3). We confirmed that the particle velocities at the bottom collision are higher than the initial velocities under both the AG and NG conditions. Moreover, the effect of Coriolis force for the change in velocity is smaller than the effect of gravitational acceleration, which means that the Coriolis force has little effect even on the disturbance of sedimentary state in the present experimental condition.

These observations collectively indicate that, while the apparent flow (fall) direction in the AG environment deviates from that of the NG environment with the falling time, our experiment is appropriate to investigate the dependence of gravity on the granular flow. In particular, for granular media #4–#8 including fine particles, the effects of adhesive forces on the granular flow under low-gravity conditions are interesting ongoing issues as its competition with gravity affects the flow-mode transitions, such as mass/funnel flows or bridging. The challenge is to develop additional experimental methods that can also study granular flow and sedimentation when adhesive forces are dominant (e.g., for very low AG).

In addition, as the particles fallen at the bottom belong to the rotating system again, it could also be possible to examine the gravity dependence on static sedimentary phenomena, including the angle of repose and bulk density. Furthermore, the data obtained by the present experiment for eight types of granular media might be used for parametric fitting and V&V^25,26^ of the DEM model under low-gravity conditions. Then, the data can be applied to simulations of more complicated dynamics of granular media and interaction phenomena with solid objects. Note that, in such future work, it is necessary to introduce a Van der Waals force and/or electrostatic force on the DEM analysis model.

### Statistical analysis

The experimental results are discussed based on Beverloo’s law^33^, which postulates the mass flow rate of granular media (${\hat{v}}_{m}$) in the steady state as

1 $${\hat{v}}_{m}={C}_{f}D{\rho }_{b}{(\xi {g}_{0})}^{0.5}$$

where *C*_*f*_ is the discharge coefficient depending on the friction coefficient of granular media, *ρ*_*b*_ is the bulk density of the granular media at the flowing part, *g*_0_ is the standard gravity acceleration (9.80665 m s^–2^), and *D* denotes the variables determined by the orifice diameter and the particle diameter. *ξ* is the gravity magnitude generated by the centrifuge; in other words, it is the scaling factor of the gravity acceleration with regard to the standard gravity acceleration.

As noted in Fig. 3, the flow rates of silica sand No. 5 and Toyoura sand are almost equivalent, although with varying particle sizes and distributions. The median diameter D50 of Silica sand No. 5 is 0.645 mm and that of Toyoura sand is 0.262 mm. Their bulk densities are approximately same (Supplementary Table 1), and the static friction coefficients for each are 0.22 and 0.18 (Supplementary Table 2), respectively. Assuming that *C*_*f*_ in Eq. (1) is proportional to the static friction coefficient, the values of the coefficient in Beverloo’s law *C*_*f*_*Dρ*_*b*_ for silica sand No. 5 and Toyoura sand have a difference of approximately 4.6%, which is almost equivalent to the difference in the flow rates of these two materials.

**Fig. 7:**
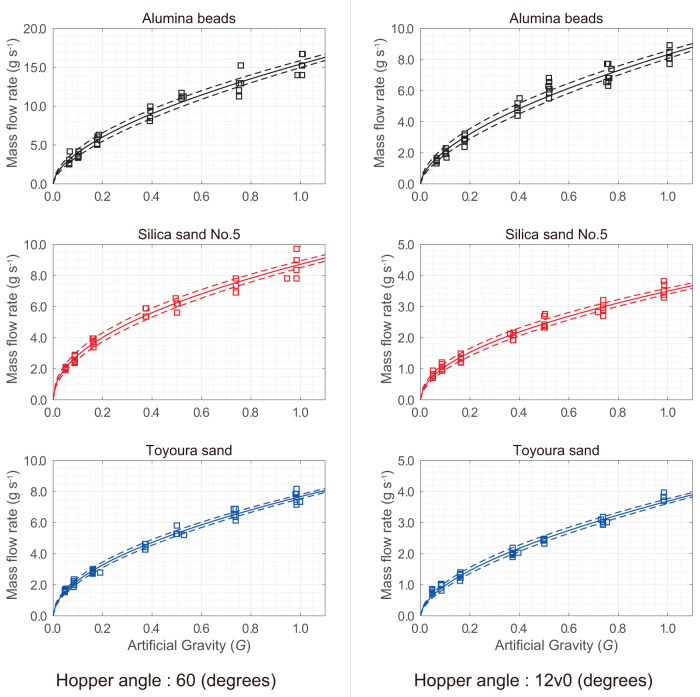
Regression analysis of the mass flow rate. The fitting curves for each graph are given based on Eq. (2) with the values of the coefficients summarised in Table 2. The regression curves have upper/lower bounds indicating a confidence level of 95% in the regression analysis.

Figure 7 summarises the regression analysis results of the mass flow rate. The regression curve is based on the nonlinear regression function as follows:

2 $${v}_{m}=\alpha {\xi }^{0.5+\beta }$$

When comparing Eqs. (1) and (2), the coefficient *α* is constant regardless of the gravity level, namely $\alpha ={C}_{f}D{\rho }_{b}{g}_{0}^{0.5}$, indicating that its value depends on the particle shape, diameter, density, and frictional property of the granular media. The coefficient *β* indicates how the experimental result deviates from the rule of the square root of gravity. Table 2 summarises the values of these coefficients determined from the analysis, which focused on the gravity magnitudes in the range of 0.0–1.0 *G* because we have sufficient datasets in those ranges. The coefficients of determination are approximately 0.9687 or higher.

**Table 2:** Coefficients of the regression curves of the mass flow rate. Here, *R*^2^ is the coefficient of determination of the regression curve. The values in the parentheses for each coefficient are the *p*-values obtained from the *F*-test of the nonlinear regression (*p < 0.05, **p < 0.01).

Hopper angle	60 °		
Granular media	$\alpha$ (g/s)	$\beta$	${R}^{2}$
Alumina beads	15.41 (0.0)**	0.0836 (1.63 × 10^−4^)**	0.972
Silica sand No. 5	8.73 (0.0)**	−0.0178 (0.222)	0.977
Toyoura sand	7.67 (0.0)**	0.0289 (0.014)*	0.989

Hopper angle	120 °		
Granular media	$\alpha \,$(g/s)	$\beta$	${R}^{2}$
Alumina beads	8.32 (0.0)**	0.0909 (1.16$\times {10}^{-4}$)**	0.969
Silica sand No. 5	3.50 (0.0)**	0.0144 (0.387)	0.975
Toyoura sand	3.69 (0.0)**	0.0770 (3.87$\times {10}^{-6}$)**	0.986

Further outlook can be discussed based on the deviation between the experiment and Beverloo’s law due to the value of *β*. Equations (1) and (2) can be combined as follows:

3 $${v}_{m}={C}_{f}D{\rho }_{b}{(\xi {g}_{0})}^{0.5}\cdot {\xi }^{\beta }$$

Here, the last term *ξ*^*β*^ can be assumed as the correction coefficient for the law. For a positive *β*, the coefficient becomes larger as the scale factor for the gravity acceleration increases. Accompanying the bulk density to the correction coefficient, namely ${\rho }_{b}^{{\prime} }={\rho }_{b}{\xi }^{\beta }$, implies that the corrected bulk density ${\rho }_{b}^{{\prime} }$ decreases as the gravitational acceleration decreases. This assumption intuitively agrees with the fact that the granular media under lower gravity acceleration falls with larger void spaces, resulting in a smaller bulk density of granular media. In other words, the porosity of the granular media increases under lower gravity accelerations. Therefore, the correction coefficient *ξ*^*β*^ may be assumed as a gravity-dependent characteristic for the bulk density of the granular media.

We calculated the *p*-values of each coefficient using the *F*-test of the nonlinear regression analysis (Table 2). The *p*-values of *β* are significant on the Alumina beads and Toyoura sand, while the value is not significant on silica sand No. 5. This may be because the particle diameter of silica sand No. 5 is approximately three times larger than those of the other two granular media (see Supplementary Fig. 1), which in turn significantly increases the gravitational force relative to the adhesive force (i.e., Van der Waals force as discussed in Fig. 2) of the unit particle of silica sand No. 5. Therefore, the flow rate of silica sand No. 5 barely requires the correction coefficient.

The abovementioned phenomenon will be useful for the design and analysis of the space probe. For example, the bulk density used in the mechanical interaction model or simulation needs to be tuned such that the density decreases as the gravity decreases.

## Methods

### Materials

In this experiment, the eight types of granular media shown in Table 1 were used. Alumina beads were adopted as the comparative material, which has almost-spherical particles and a considerably narrow particle size distribution. To enable an image analysis, 30% of packed alumina beads were coloured black using a permanent marker. In addition, as typical sandy soils for which many soil test results have been reported, silica sand (Tohoku sand) No. 5 and No. 8 and Toyoura sand were adopted^45–47^. Furthermore, considering the use for future space exploration, four types of regolith simulants, including Lunar regolith simulant^31^ and Phobos regolith simulant^48^, were adopted. The details of the soil test results, such as regarding the particle size distribution, shear strength obtained using a direct shear test, particle and bulk density tests, and static friction test, are summarised in Supplementary Methods.

### Experimental apparatus

The experimental apparatus consists of the hourglass-shaped device, CPU board with a camera module, servo motor, accelerometer, and battery packs whose specifications are listed in Supplementary Methods. The hourglass is composed of aluminium alloy, and its front and back sides are covered by borosilicate glass. The hourglass packs a certain amount of granular material in vacuum of less than 20 Pa. The hourglass has two different hopper angles (60° and 120°). The servo motor flips the hourglass 180° with respect to the artificial gravitational direction every minute so that we can observe granular flow from the different hopper angles in every hourglass flip, where the flip takes apploximately 0.6 s to complete. Note that the rotation range (flipping motion) is limited to ±90° because of the size constraints of the experimental apparatus.

The CPU with the onboard camera grabs the flow images at 25 fps and stores them in a compressed MP4 format (H.264, 40 Mbps) file every five minutes. These images are resampled from MP4 files at a constant frame rate and undistorted using a calibration template calculated at the pre-flight check. Each image is then correlated with the gravity condition generated by the AG.

The mass flow rate *v*_*m*_ is calculated from the experiment as:

4 $${v}_{m}=\frac{m}{{t}_{e}-{t}_{0}}$$

where *m* is the mass of the granular media packed in the hourglass; *t*_0_ is the time when the flow starts, which is equivalent to the moment when the hourglass is perpendicular to the AG direction during its flip; and *t*_*e*_ is the time when particles do not move among the consecutive images. The temporal resolution is 0.04 s, which is determined by the camera frame rate (25 fps).

### Discrete element method

The DEM is a method in which the analytical object is modelled as an aggregate of discrete particles, and their motions at each instant is analysed considering contact and sliding between the particles or particle and object^17,18^. In this study, the commercial software package Rocky DEM^49^ was used.

In the analyses, each granular media was represented by an aggregate of spherical particles according to their particle size distributions (see Supplementary Fig. 1). The Hertzian spring-dashpot and Mindlin-Deresiewicz models were adopted for the normal and tangential force models, respectively. Further, to express the effect of actual particle shape, the rolling resistance model (type C) was also introduced^50^. Rolling resistance is considered when a moment opposing the rolling motion of a particle is introduced into the modelling. This moment is usually incorporated for practical representation of the effect of non-sphericity on rolling spheres or the effect of surface irregularities on other types of particles. The rolling resistance contributes to the dissipation of energy during relative rotation, which is important for dynamic flow conditions. In this study, we focused on the dynamic flow of granular media that are relatively sparse; therefore, we incorporated the effects of sand particle shapes using the rolling resistance model. Notably, the particle shape is important in the DEM analysis for problems with prominent dilatancy^51^. To thoroughly examine the relationship between dilatancy trends, bulk density, and gravity of granular media except for alumina beads in the future, it is necessary to newly identify the parameters of the DEM model using non-spherical particles.

Table 3 shows the parameters of alumina beads, silica sand No. 5, and Toyoura sand for the present analysis. Here, these parameters were determined with reference to the results of minimum density test and the residual strength obtained by a direct shear test, in addition to the hourglass experiment. Friction coefficients between granular media and diagonal wall of hourglass were obtained using a static friction test (see Supplementary Table 2).

**Table 3:** Parameters of Alumina beads, Silica sand No. 5, and Toyoura sand for DEM analysis. Here, to reproduce the size distributions of the granular media in the DEM analysis, a histogram of the cumulative mass% for each particle size was created based on sieving interval. The sieving interval corresponds to the cumulative mass distribution data (see Data Availability section). Particle sizes are generated according to their proportions using the function of Rocky DEM^49^.

Particle	Alumina beads	Silica sand No. 5	Toyoura sand
Density (g/cm^3^)	3.827	2.648	2.663
Young’s modulus (MPa)	500	500	500
Poisson’s ratio (-)	0.3	0.3	0.3
Rolling resistance (-)	0.01	0.3	0.3
Particle–particle			
Static friction coefficient (-)	0.30	0.50	0.50
Dynamic friction coefficient (-)	0.30	0.50	0.50
Restitution coefficient (-)	0.30	0.30	0.30
Particle–hourglass			
Static friction coefficient (-)	0.33	0.22	0.18
Dynamic friction coefficient (-)	0.33	0.22	0.18
Restitution coefficient (-)	0.30	0.30	0.30

The analysis assuming the NG environment was performed by setting a uniform gravity field and giving only flips to the hourglass. Under this condition, a uniform gravity field such as found on Earth and other planets was reproduced. Meanwhile, in the analysis assuming the AG environment at the ISS, in addition to the flips, revolution corresponding to the rotary table was given to the hourglass under the boundary condition of a zero-gravity field. Under this condition, the adjusted centrifugal force acting on the granular media in the inward volume of the hourglass was reproduced. Thus, once the particles pass through the orifice, the material experiences zero-gravity (whereas for the experiment, the particle experiences only ISS microgravity). Here, the centre and radius of the evolution and the respective angular velocities were set to the same values as those in the experiment (see Supplementary Fig. 2). Regarding the influence of the AG gradient and selection of the location of the radial position, we confirmed that the effects are minor in the range of present experimental geometry (see supplementary Table 5). Note that, for the analysis in Fig. 5, the gravitational accelerations under NG conditions were adjusted to the gravity at the centre of the orifice under AG conditions.

### Reporting summary

Further information on research design is available in the Nature Research Reporting Summary linked to this article.

### Supplementary information

Supplementary Information

Supplementary Movies 1

Supplementary Movies 2

Supplementary Movies 3

Reporting Summary

## Data Availability

The authors confirm that the data supporting the findings of this study are available within the supplementary materials. Further, the representative MP4 data and raw data including soil properties are available via the Open Science Framework at https://osf.io/3zcm2/.
