# Publisher Correction: Granular flow experiment using artificial gravity generator at International Space Station

**DOI:** 10.1038/s41526-023-00325-9

**Published:** 2023-09-22

**Authors:** S. Ozaki, G. Ishigami, M. Otsuki, H. Miyamoto, K. Wada, Y. Watanabe, T. Nishino, H. Kojima, K. Soda, Y. Nakao, M. Sutoh, T. Maeda, T. Kobayashi

**Affiliations:** 1 Yokohama National University, Yokohama, Japan; 2 Keio University, Yokohama, Japan; 3 Japan Aerospace Exploration Agency, Sagamihara, Japan; 4 The University of Tokyo, Tokyo, Japan; 5 Chiba Institute of Technology, Chiba, Japan; 6 Tokyo University of Agriculture and Technology, Fuchu, Japan; 7 Ritsumeikan University, Kusatsu, Japan

**Keywords:** Aerospace engineering, Mechanical engineering

Correction to: *npj Microgravity* 10.1038/s41526-023-00308-w, published online 08 August 2023

The updated Table 1 with a comment indicating that micrographs #05 and #08 at the bottom of the images should be replaced. However, in the response to author query 4, Micrographs #4 and #8 were changed in Table 1. While processing the suggested changes based on the eProofing comments, the correction team updated the existing table figures and replaced image #05 with the micrograph of image #08 and image #08 with the micrograph of image #04 in the revised table. As a result, the changes got reverted and images were incorrect and duplicated.

