# Supplementary material for: Granular flow experiment using artificial gravity generator at International Space Station
Source: NPJ Microgravity. 2023 Aug 8;9:61. doi: 10.1038/s41526-023-00308-w (PMC10409782; doi:10.1038/s41526-023-00308-w)
Supplement: Supplementary file 1 — Supplementary Information [file 41526_2023_308_MOESM1_ESM.pdf]

Supplementary information

Granular flow experiment using artificial gravity generator at International Space Station

S. Ozaki[1*], G. Ishigami[2+], M. Otsuki[3+], H. Miyamoto[4], K. Wada[5], Y. Watanabe[1], T. Nishino[1], H. Kojima[2], K. Soda[2], Y. Nakao[2], M. Sutoh[3], T. Maeda[6], and T. Kobayashi[7]

[1]Yokohama National University, [2]Keio University, [3]Japan Aerospace Exploration Agency, [4]The University of Tokyo, [5]Chiba Institute of Technology, [6]Tokyo University of Agriculture and Technology, [7]Ritsumeikan University, [+]equally contributed.

[1*] 79-5 Tokiwadai, Hodogaya-ku, Yokohama 240-8501, Japan. Email: s-ozaki@ynu.ac.jp

[2+] 3-14-1 Hiyoshi, Yokohama 223-8522, Japan. Email: ishigami@mech.keio.ac.jp.

[3+] 3-1-1 Yoshinodai Chuo-ku, Sagamihara-shi, Kanagawa 252-5210, Japan. Email: otsuki.masatsugu@jaxa.jp

Corresponding author: S. Ozaki

Yokohama National University

79-5 Tokiwadai, Hodogaya-ku, Yokohama 240-8501, Japan

Phone: +81-45-339-3881

Fax: +81-45-339-3881

Email: s-ozaki@ynu.ac.jp

Supplementary Notes

The DEM analysis results of alumina beads and silica sand No. 5 under 0.1 *G* are shown in supplementary movies 1 and 2. In the NG environment, the prescribed uniform gravity field was set, and only flips were imposed on the hourglass. Meanwhile, in the AG environment, both the revolution corresponding to the rotary table and the flips were imposed on the hourglass under the boundary condition of a zero-gravity field. Here, the centre and radius of revolution and respective angular velocities were the same as those of the experiment. Note that the video is played at 0.5x speed.

The comparison between the experiment and DEM analysis results of alumina beads under 1.0 *G* (NG) is shown in the supplementary movie 3. This experiment was performed on Earth. The granular flow behaviour under 1.0 *G* (NG) was accurately reproduced under the present DEM analysis conditions. The average mass flow rates (60°, 120°) obtained from the experiment and DEM were (15.0, 11.5) g s^{-1} and (14.9, 9.60) g s^{-1}, respectively. Note that the video is played at 0.2× speed.

Supplementary Methods

This supplementary methods contains five parts. First, we introduce detailed information of the granular media used in the experiment. Next, we outline the layout of the experimental apparatus, including the geometric information of the hourglass and centrifuge. Then, we describe the specifications of the experimental components along with those of the sensor and camera, and also describe the acceleration histories at each AG condition. Finally, we provide the method for measuring particle velocity based on DEM analysis.

In this experiment, the eight types of granular media shown in Table 1 were used. The alumina beads were adopted as a comparative material which has a nearly spherical shape and quite narrow particle size distribution. In addition, silica sand (Tohoku sand)

No. 5 and No. 8 and Toyoura sand were adopted as typical sandy soils for which many soil tests results have been reported[1-3]. In particular, the latter is often used as standard sand in soil mechanics[1]. Furthermore, considering the use for future space exploration, four types of regolith simulants—specifically, Lunar regolith simulant (FJS-1)[4], Phobos regolith simulant[5], and Martian regolith simulant (JSC MARS-1A)—were adopted.

The details of the soil test results, such as particle size distribution, particle density and bulk density tests, direct shear test, and static friction test, are summarised below.

Supplementary Figure 1 shows the particle size distribution of each granular media, which was measured by the laser diffraction particle size analyser (SALD-2300, SHIMADZU Co.). Supplementary Table 1 shows the particle densities measured according to JIS A 1202: 2009 (ISO 17892-3:2015) and the maximum and minimum bulk densities measured according to JIS 1224: 2009.

All granular media were prepared via the following process: first, the granular media were dried for 24 hours at 120 °C, and then sieved at 850 μm (425 μm only for #04 FSJ-1). Subsequently, the granular media were packed into the hourglass and vacuumed. We confirmed that the hourglass maintains the vacuum level in the range of 15–20 Pa after 2 hours followed by sealing the hourglass with the resin packing. Note that the air may gradually return to the hourglass at the moment of the experiment because it took approximately seven months from the sealing to the experiment (handover, launch, and delivery to the ISS. However, we estimate that the air pressure of the hourglass at the moment of the experiment is still less than approximately 30 Pa based on the specification of the vacuum valve implemented on the hourglass.

Strength parameters were obtained from constant-pressure direct shear tests (JGS 0561-2009). The internal friction angle ϕ_d and cohesion c_d were evaluated under three

levels of relative density conditions, i.e., 50, 70, and 90%. The raw data are available via the Open Science Framework at https:/osf.io/3zcm2/.

The frictional property between particles and the diagonal wall of the hourglass is one of the most important information for analysing granular flow characteristics. The static friction coefficient measured by the static friction test apparatus (TRIBOGEAR TYPE:10, SHINTO Scientific Co.) and shown in Supplementary Table 2 is used as both static and dynamic friction coefficients in DEM analysis.

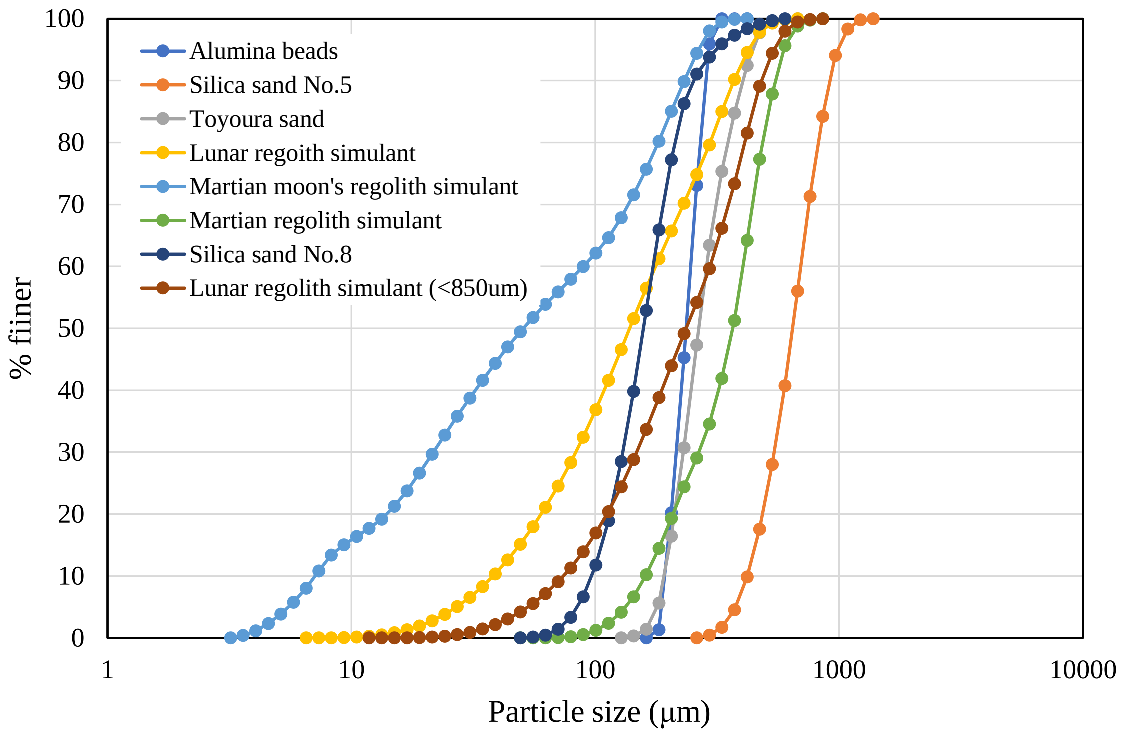

Supplementary Figure 1: Particle size distribution of eight types of granular media. The raw data are available via the Open Science Framework at https:/osf.io/3zcm2/.

Supplementary Table 1: Particle and bulk densities data. Each particle density is the average of three samples, while each bulk density is the average of five samples.

#	Name	Particle density (g/cm^3)	Bulk density (g/cm^3)	
			Maximum	Minimum
1	Alumina beads	3.819	2.349	2.116
2	Silica sand No. 5	2.701	1.699	1.467
3	Toyoura sand	2.661	1.631	1.378
4	Lunar regolith simulant (FJS-1, sieved)	2.899	1.908	1.497
5	Phobos regolith simulant	2.907	1.352	1.056
6	Martian regolith simulant	2.636	0.940	0.742
7	Silica sand No. 8	2.634	1.511	1.169
8	Lunar regolith simulant (FJS-1, original)	2.890	1.979	1.551

Supplementary Table 2: Static friction coefficients between particles and diagonal wall of the hourglass. These values are each the average value obtained from respective five samples.

#	Name	Static friction coefficient
1	Alumina beads	0.33
2	Silica sand No. 5	0.22
3	Toyoura sand	0.18
4	Lunar regolith simulant (FJS-1, sieved)	0.25
5	Phobos regolith simulant	0.30
6	Martian regolith simulant	0.25
7	Silica sand No. 8	0.27
8	Lunar regolith simulant (FJS-1, original)	0.30

The centrifuge on the ISS mounts four sets of the experimental apparatus on its turntable (Supplementary Figure 2). The centrifuge can produce artificial gravity in the range of 0.063–2.0 *G* by controlling the angular velocity of the turntable ω_c that can geometrically be determined by the following equation:

$$\omega_c = \sqrt{\frac{\hat{g}_{AG}}{r_0}} \tag{1}$$

where $\hat{g}_{AG}$ is the reference AG and r_0 is the radius measured from the rotation centre of the turntable to the reference point of the experimental apparatus (P in Supplementary Figure 2). Supplementary Table 3 lists the relationship between the reference AGs in our experiment and centrifuge angular velocity. Owing to the centrifuge principle, the AG at the position r_{xyz} inside the hourglass with respect to the reference P varies according to the following equation:

$$g_{xyz} = \frac{r_{xyz}}{r_0}\hat{g}_{AG} \tag{2}$$

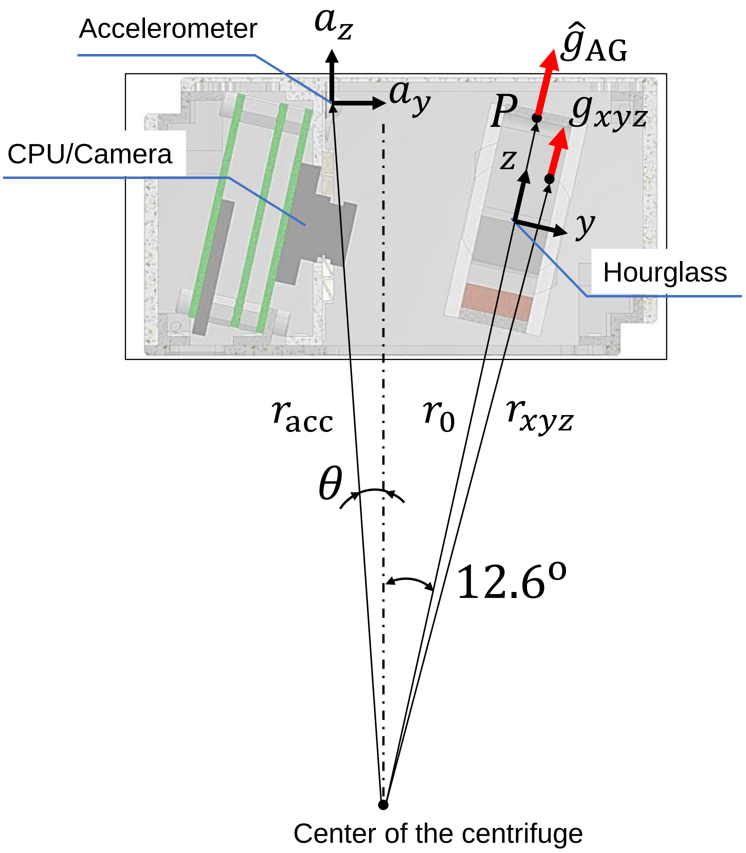

Supplementary Figure 2: Geometric information of the experimental apparatus on the centrifuge. r_0 is 139.33 mm.

Supplementary Table 3: Relationship between the reference AG and the rotational angular velocity of the centrifuge.

$\hat{g}_{AG}$ [G]	ω_c [rpm]
0.063	20.1
0.10	25.3
0.17	33.0
0.38	49.4
0.50	56.7
0.75	69.4
1.00	80.1
2.00	113.1

The hourglass is mounted on the apparatus with an offset tile angle of 12.6° to make the hourglass central axis coincide with the AG direction (Supplementary Figure 2).

The components used in the experimental apparatus include a CPU board with a camera module, accelerometer, servo motor, and battery packs (Supplementary Figure 3 (a)). The hourglass is composed of aluminium alloy, and its front and back sides are covered by borosilicate glass. The dimensions of hourglass are shown in Supplementary Figure 3(b).

The CPU board with a camera module, or main control unit (MCU), is Armadillo 810 (Atmark Techno Inc.) running a Linux OS dedicated to the MCU. Supplementary Figure 4 shows the diagram of the MCU and peripheral devices. The camera is mounted in a position where the entire hourglass can be captured. It has a rolling shutter image sensor with a resolution of 1280 × 960 and frame rate of 25 fps. Notably, the size, reliability, and power available from the experimental apparatus limited us to using only this modest camera module. The optical axis of the camera is perpendicular to the vertical axis of the hourglass. The field of view of the camera is 119 × 99°, which leads to significant image distortion and is calibrated using the Computer Vision Toolbox of MATLAB®. The SD card mounted on the extension board stores all the MP4 files

captured by the camera and acceleration data in the experiment. After the experiments were completed, the astronaut on the ISS supporting our experiment ejected the SD cards from each apparatus. Some MP4 files were directly downlinked from the ISS to a ground station for quick-look purposes, while others were sent back to Earth.

The MEMS accelerometer (Analog Devices ADXL355) measures the three-axis acceleration at 50 Hz. The acceleration measured by the sensor and the AG at the reference point (P in Supplementary Figure 2) differ because of the geometric offset. Therefore, the measured data must be converted to that of the acceleration on the hourglass:

$$g_{AG} = \left(a_y \cos\theta - a_z \sin\theta\right) r_0 / r_{acc} \tag{3}$$

where g_{AG} is the AG at the reference point of the hourglass (Supplementary Figure 2), a_y and a_z are the accelerations measured at the sensor coordinates, θ is the offset angle of the *x*-*y* plane relative to the AG direction, and r_{acc} is the distance between the sensor and the centre of the centrifuge.

(a)

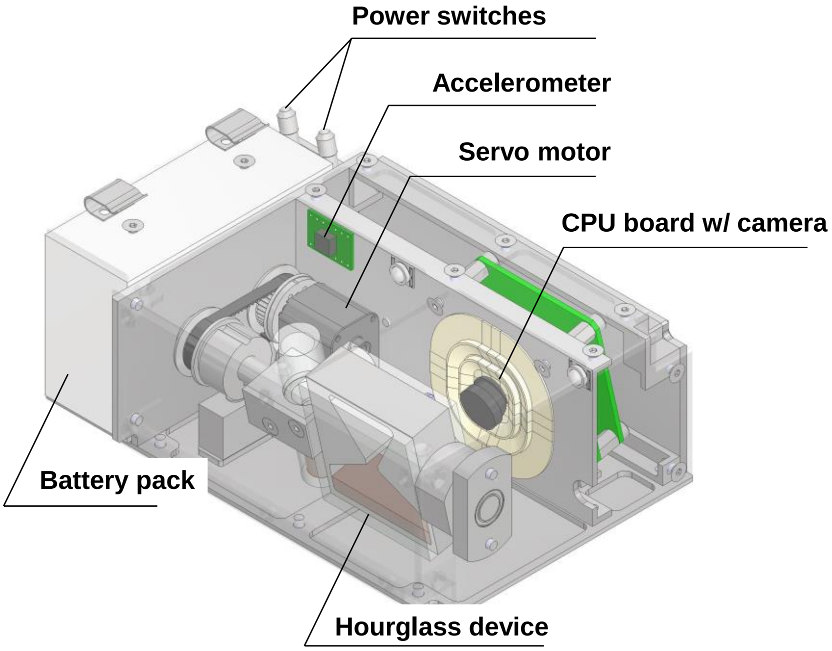

(**b**)

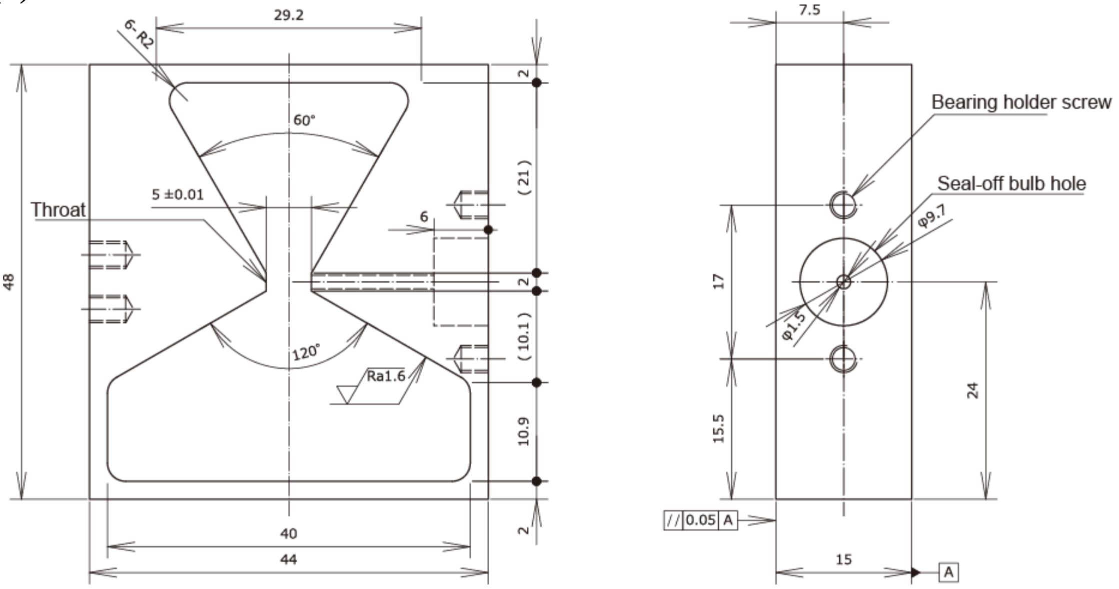

Supplementary Figure 3: Schematic illustration of the experimental apparatus: (**a**) Component layout of the apparatus; (**b**) Specifications of hourglass shape (unit: mm).

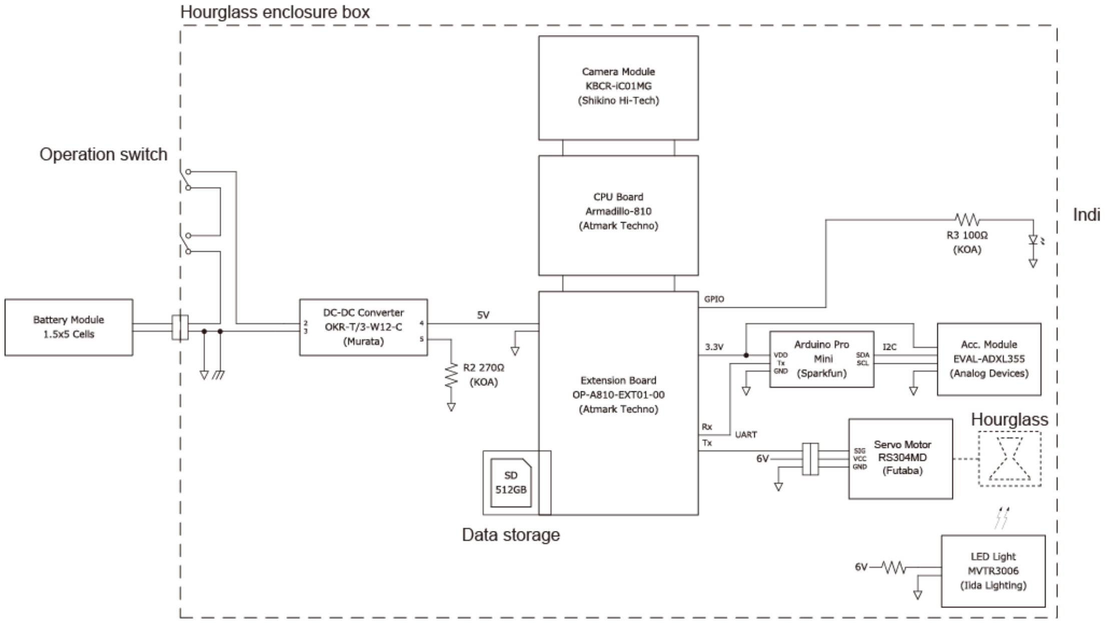

Supplementary Figure 4: System diagram of the main control unit and peripheral devices. (The figures are currently compressed, they will be replaced with higher-resolution versions for the submission.)

The comparison of the statistical data of AG with the reference gravity level is summarised in Supplementary Table 4. The centrifuge accurately produces the AG within a difference of 0.02 G. The standard deviation of the AG is also negligible. Therefore, the experimental campaign was completed under high-quality AG conditions.

The AG profile for each reference gravity level is summarised in Supplementary Figure 5. The periodic vibration of the raw acceleration data (blue-coloured lines) is due to the mechanical misalignment of the centrifuge as well as the resolution of the motor controller for the centrifuge. Further, the spike noise observed every 60 s is due to the flip motion of the hourglass.

Supplementary Table 4: Statistics of AG in the experiment.

Reference $\hat{g}_{AG}$ (G)	Generated g_{AG} (Ave.) (G)	Generated g_{AG} (Std.) (G)
0.063	0.067	0.009
0.100	0.103	0.013
0.170	0.180	0.021
0.380	0.393	0.029
0.500	0.519	0.008
0.750	0.758	0.008
1.00	1.01	0.006
2.00	1.98	0.006

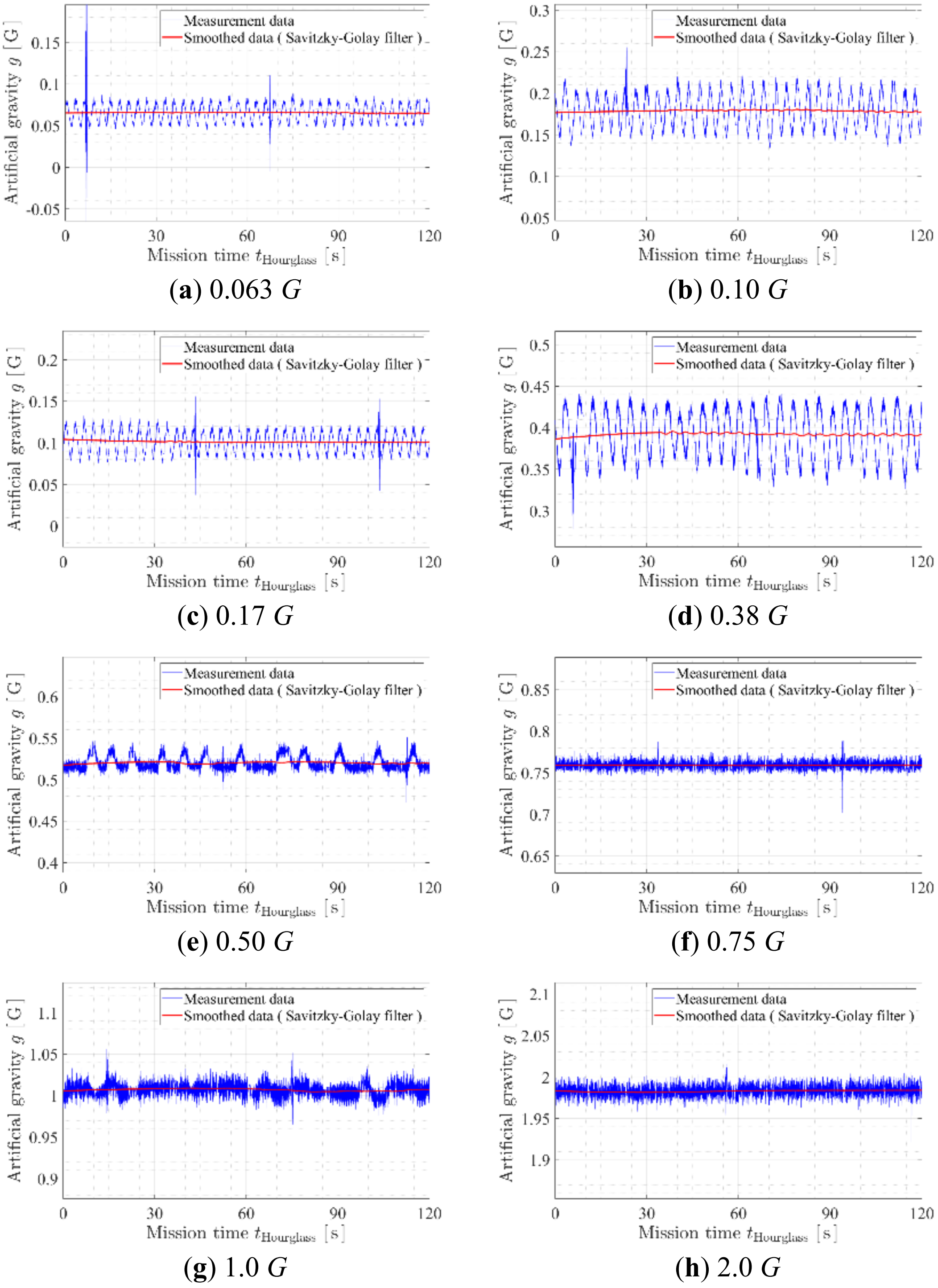

Supplementary Figure 5: AG profiles generated by the centrifuge.

As shown in Supplementary Figure 6, for both the NG and AG conditions, the particle velocity shown in Figures 5 and 6 was measured in a cuboid region at the centre position immediately below the orifice. The size of the cuboid region is 5 mm×15 mm in the *x*-*y* plane and it has a length of 1 mm in the *z* direction (longitudinal direction). The particle velocity was evaluated as the average velocity value of all particles that passed through the cuboid region. Note that the velocity was different at the beginning and end of the flow. Detailed data regarding the three granular media are available via Open Science Framework at https:/osf.io/3zcm2/. Here, the gravitational accelerations under the NG conditions were adjusted to the gravity at the centre of the orifice under the AG conditions. Therefore, while the particles were passing through the orifice, which was the measurement position of particle velocity shown in Supplementary Figure 6, the AG acting on the particles had the same magnitude of force as that of the NG (0.053, 0.084, 0.25, 0.42, 0.59, and 0.84 *G*, respectively).

Furthermore, to examine the influence of the AG gradient due to the radial distance from the centre of the centrifuge, we performed a DEM analysis. In this analysis, we set two radial positions to create the prescribed centrifugal force at the orifice and bottom of the hourglass. Here, the target AG values were 0.063 and 1.0 *G*. Supplementary Table 5 lists the mass flow rates of alumina beads obtained under each condition. The relative difference in AG between these two cases was 16%, that is, the absolute differences were 0.01 *G* and 0.16 *G* for 0.063 *G* and 1.0 *G*, respectively. The difference in mass flow rate due to the target radial position of AG was smaller than the dissipation of the measured values in the experiment (see Figure 3). It is implied that the effects of the AG gradient on mass flow rate are minor in the range of the present experimental geometry, especially under low gravity conditions.

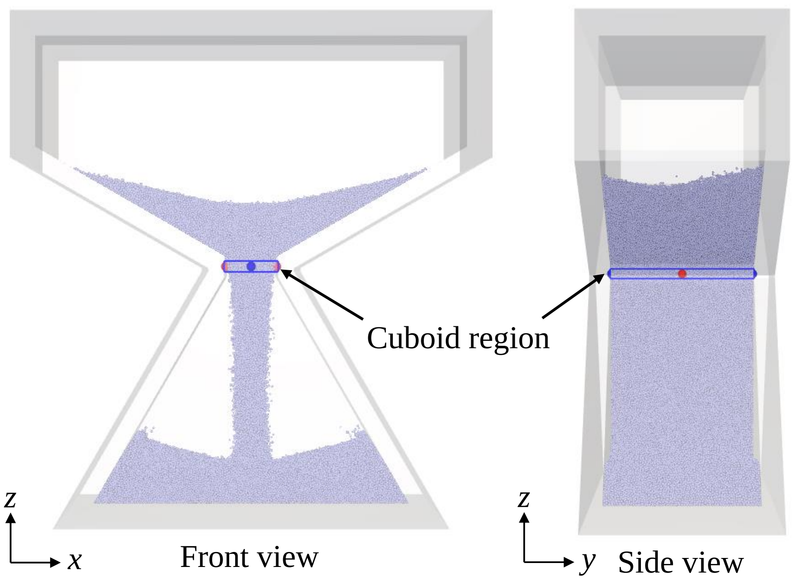

Supplementary Figure 6: Measurement position of particle velocity. The size of the cuboid region is 5 mm × 15 mm in the *x*-*y* plane and it has a length of 1 mm in the *z* direction.

Supplementary Table 5: DEM analysis results. Effect of AG gradient on mass flow rate of alumina beads.

**Target radial position**	**Bottom** (Experimental condition at ISS)	**Centre of orifice**
Mass flow rate (60°) (0.063 *G*)	4.7 g s^{-1}	5.1 g s^{-1}
Mass flow rate (60°) (1.0 *G*)	14.9 g s^{-1}	15.4 g s^{-1}
Mass flow rate (120°) (0.063 *G*)	3.0 g s^{-1}	3.3 g s^{-1}
Mass flow rate (120°) (1.0 *G*)	9.6 g s^{-1}	10.4 g s^{-1}

Supplementary References

1. Iwasaki, T., Tatusoka, F. & Takagi, T. Shear moduli of sands under cyclic torsional shear loading. *Soils Found.* **18**, 39–56 (1978).
2. Oztoprak, S. & Bolton, M.D. Stiffness of sands through a laboratory test database. *Geotechnique* **63**, 54–70 (2013).

3. Arshad, M.I., Tehrani, F.S., Prezzi, M. & Salgado, R. Experimental study of cone penetration in silica sand using digital image correlation. *Geotechnique* **64**, 551–569 (2014).
4. Kobayashi, T., Fujiwara, Y., Yamakawa, J. & Yasufuku, N. Mobility performance of a rigid wheel in low gravity environments. *J. Terramech*. **47**, 261–274 (2010).
5. Miyamoto, H. et al. Surface environment of Phobos and Phobos simulant UTPS. *Earth Planets Space* **73**, 214 (2021).
