# Supplementary material for: Granular flow experiment using artificial gravity generator at International Space Station
Source: NPJ Microgravity. 2023 Aug 8;9:61. doi: 10.1038/s41526-023-00308-w (PMC10409782; doi:10.1038/s41526-023-00308-w)
Supplement: Supplementary file 5 — Reporting Summary [file 41526_2023_308_MOESM5_ESM.pdf]

nature portfolio

Corresponding author(s): Shingo Ozaki, NPJMGRAV-00995R

Last updated by author(s): Jul 4, 2023

Reporting Summary

Nature Portfolio wishes to improve the reproducibility of the work that we publish. This form provides structure for consistency and transparency in reporting. For further information on Nature Portfolio policies, see our Editorial Policies and the Editorial Policy Checklist.

Statistics

For all statistical analyses, confirm that the following items are present in the figure legend, table legend, main text, or Methods section.

n/a	Confirmed	
☐	☒	The exact sample size (*n*) for each experimental group/condition, given as a discrete number and unit of measurement
☐	☒	A statement on whether measurements were taken from distinct samples or whether the same sample was measured repeatedly
☐	☒	The statistical test(s) used AND whether they are one- or two-sided<br>*Only common tests should be described solely by name; describe more complex techniques in the Methods section.*
☐	☒	A description of all covariates tested
☐	☒	A description of any assumptions or corrections, such as tests of normality and adjustment for multiple comparisons
☐	☒	A full description of the statistical parameters including central tendency (e.g. means) or other basic estimates (e.g. regression coefficient) AND variation (e.g. standard deviation) or associated estimates of uncertainty (e.g. confidence intervals)
☐	☒	For null hypothesis testing, the test statistic (e.g. *F*, *t*, *r*) with confidence intervals, effect sizes, degrees of freedom and *P* value noted<br>*Give P values as exact values whenever suitable.*
☒	☐	For Bayesian analysis, information on the choice of priors and Markov chain Monte Carlo settings
☒	☐	For hierarchical and complex designs, identification of the appropriate level for tests and full reporting of outcomes
☒	☐	Estimates of effect sizes (e.g. Cohen's *d*, Pearson's *r*), indicating how they were calculated

Our web collection on statistics for biologists contains articles on many of the points above.

Software and code

Policy information about availability of computer code

Data collection	N/A
Data analysis	Commercial software package RockyDEM was used for DEM analysis. The non-linear regression analysis was done by the Statistics and Machine Learning Toolbox for MATLAB.

For manuscripts utilizing custom algorithms or software that are central to the research but not yet described in published literature, software must be made available to editors and reviewers. We strongly encourage code deposition in a community repository (e.g. GitHub). See the Nature Portfolio guidelines for submitting code & software for further information.

Data

Policy information about availability of data

All manuscripts must include a data availability statement. This statement should provide the following information, where applicable:

- Accession codes, unique identifiers, or web links for publicly available datasets
- A description of any restrictions on data availability
- For clinical datasets or third party data, please ensure that the statement adheres to our policy

The authors confirm that the data supporting the findings of this study are available within the supplementary materials. Further, the representative MP4 data and raw data including soil properties are available via the Open Science Framework at https:/osf.io/3zcm2/.

Research involving human participants, their data, or biological material

Policy information about studies with human participants or human data. See also policy information about sex, gender (identity/presentation), and sexual orientation and race, ethnicity and racism.

Reporting on sex and gender	N/A
Reporting on race, ethnicity, or other socially relevant groupings	N/A
Population characteristics	N/A
Recruitment	N/A
Ethics oversight	N/A

Note that full information on the approval of the study protocol must also be provided in the manuscript.

Field-specific reporting

Please select the one below that is the best fit for your research. If you are not sure, read the appropriate sections before making your selection.

☐ Life sciences ☐ Behavioural & social sciences ☒ Ecological, evolutionary & environmental sciences

For a reference copy of the document with all sections, see nature.com/documents/nr-reporting-summary-flat.pdf

Ecological, evolutionary & environmental sciences study design

All studies must disclose on these points even when the disclosure is negative.

Study description	We performed the experiment to examine a granular flow under stable artificial gravity conditions for a long duration generated by a centrifuge at the International Space Station. Experimental units is eight (the eight types of granular media).
Research sample	In this experiment, the eight types of granular media. Alumina beads were adopted as the comparative material, which has almost-spherical particles and a considerably narrow particle size distribution. In addition, as typical sandy soils for which many soil test results have been reported, silica sand (Tohoku sand) No. 5 and No. 8 and Toyoura sand were adopted. Furthermore, considering the use for future space exploration, four types of regolith simulants, including Lunar regolith simulant and Phobos regolith simulant, were adopted.
Sampling strategy	The mass of the granular media m were determined by considering the total volume of the narrower space of the hopper and the minimum bulk density. The value of mass is conservatively determined to avoid the overflow of the granular media.
Data collection	The SD card mounted on the test apparatus stores all the MP4 files captured by the camera and acceleration data in the experiment. After the experiments were completed, the astronaut on the ISS supporting our experiment ejected the SD cards from each apparatus. Some MP4 files were directly downlinked from the ISS to a ground station for quick-look purposes, while others were sent back to Earth.
Timing and spatial scale	The sample was launched by H-IIB rocket No. 8 on September 25, 2019, and operated on the ISS for one day each in February and May 2020.The experimental campaign continued for seven hours. Each hourglass continued flipping every 60 s to change the flow direction to different hopper angles (60° and 120°). The total number of flipping was more than 400 during the experimental campaign.
Data exclusions	There is no data were excluded from the analyses.
Reproducibility	The experimental campaign continued for seven hours. Each hourglass continued flipping every 60 s to change the flow direction to different hopper angles (60° and 120°). The total number of flipping was more than 400 during the experimental campaign. The reproducibility of the experiment was confirmed from the videos taken over the entire campaign period.
Randomization	This is not relevant to our study because we used well-homogenized granular media as samples.
Blinding	This is not relevant to our study.

Did the study involve field work? ☐ Yes ☒ No

Reporting for specific materials, systems and methods

We require information from authors about some types of materials, experimental systems and methods used in many studies. Here, indicate whether each material, system or method listed is relevant to your study. If you are not sure if a list item applies to your research, read the appropriate section before selecting a response.

Materials & experimental systems

n/a	Involved in the study
☒	☐ Antibodies
☒	☐ Eukaryotic cell lines
☒	☐ Palaeontology and archaeology
☒	☐ Animals and other organisms
☒	☐ Clinical data
☒	☐ Dual use research of concern
☒	☐ Plants

Methods

n/a	Involved in the study
☒	☐ ChIP-seq
☒	☐ Flow cytometry
☒	☐ MRI-based neuroimaging
